# Lower, Variable Intrathecal Opioid Doses, and the Incidence of Prolonged Fetal Heart Rate Decelerations After Combined Spinal Epidural Analgesia for Labor: A Quality Improvement Analysis

**DOI:** 10.2478/rjaic-2020-0015

**Published:** 2020-12-31

**Authors:** Sheena Hembrador, Carlos Delgado, Emily Dinges, Laurent Bollag

**Affiliations:** 1 Department of Anesthesiology and Pain Medicine, University of Washington, 1959 NE Pacific Street, Box 356540, Seattle, WA 98195, USA; 2 Present Address: Department of Anesthesiology, Virginia Mason Medical Center, 1100 9^th^ Ave, Seattle, WA 98101, USA

**Keywords:** Fentanyl, fetal bradycardia, combined spinal-epidural

## Abstract

**Background:**

Combined spinal-epidurals with low-dose intrathecal opioids and local anesthetics are commonly used to initiate labor analgesia due to the fast onset of analgesia and good patient satisfaction. Intrathecal fentanyl has been associated with fetal bradycardia, and the rate may be higher at doses of 25 mcg and above. As such, our institution limits intrathecal fentanyl doses to less than 15 mcg for labor. Prompted by a few incidents of prolonged fetal bradycardia at even these low doses, we sought to audit the side effects associated with varying low doses of intrathecal fentanyl.

**Methods:**

After IRB approval, a retrospective review was performed on 555 labor records from May–December, 2016. All the patients received combined spinal epidurals for labor analgesia. Intrathecal medication consisted of 1 mL of 0.25% bupivacaine, and varying fentanyl doses: 2.5, 5, 10, and 15 mcg. The incidences of prolonged fetal heart rate decelerations, emergent cesarean delivery, magnitude of pain reduction, pruritus requiring treatment, and hypotension were compared.

**Results:**

Demographic variables were equivalent between the groups. There were no differences in the rates of prolonged fetal decelerations (in order of increasing fentanyl dose: 4.4%, 2.3%, 7.6%, 3.0%, p-value = 0.11), emergent cesarean delivery, magnitude of pain reduction, pruritus, or maternal hypotension.

**Conclusions:**

In conclusion, the rates of prolonged fetal heart rate decelerations after combined spinal epidural with intrathecal bupivacaine and fentanyl does not differ for fentanyl doses of 15 mcg and below.

## Introduction

The combined spinal-epidural (CSE) technique with a intrathecal (IT) dose of local anesthetic and lipophilic opioid has become a popular method of initiating labor analgesia as it has been shown to have faster onset of analgesia with less profound motor block, and higher satisfaction ratings from patients and obstetric practitioners compared to lumbar epidurals (LEPs).^[1, 2, 3, 4, 5]^ CSEs have also been associated with less recurrent breakthrough pain than LEPs after labor analgesia initiation.[2] The advantage that CSEs have from faster onset of analgesia is slightly diminished earlier in the first stage of labor (<6 cm cervical dilation). Nevertheless, satisfaction metrics are shown to be similar in early labor.[6,7] It is for these reasons that the CSE technique is the preferred method of initiating labor analgesia at our institution.

While some maternal complications, namely postural headache, cesarean delivery, and shoulder dystocia, occur at similar rates in parturients with CSEs and LEPs,^[8, 9, 10, 11]^ pruritis occurs more frequently with CSEs,^[12, 13, 14, 15]^ and the data is mixed for non-reassuring fetal heart rate changes (NRFHRC).^[12,14,16, 17, 18, 19]^ To provide adequate analgesia while minimizing the risks of NRFHRC and pruritis from higher IT fentanyl doses, many anesthesiologists have opted to use lower doses of IT lipophilic opioids (≤25 mcg fentanyl), or a combination of low-dose lipophilic opioid and local anesthetic to take advantage of the synergistic relationship between the two. ^[18, 19, 20, 21]^ The exact dose of IT opioid at which the risk of NRFHRCs starts to climb has been studied and is yet unknown.^[3, 4,8,10,18,20, 21, 22, 23]^ It is important to note that data suggests doses of IT fentanyl up to 15 mcg, when combined with bupivacaine 2.5 mg, provide satisfactory analgesia to all parturients.[18]

Women should receive the best care possible when pregnant, and we should continuously monitor our interventions and address how to improve them. After all, quality improvement is fundamental to obstetric anesthesia practice.[24] Prompted by a small series of prolonged NRFHRC at our institution when using doses up to 15 mcg of fentanyl, we performed a quality assessment audit of normal practice variation with different IT fentanyl doses (2.5, 5, 10, and 15 mcg) combined with 2.5 mg of IT bupivacaine. Our primary outcome was the incidence of NRFHRCs. Our secondary outcomes were need for emergent cesarean delivery after neuraxial blockade placement during labor, analgesic efficacy of varying IT fentanyl doses, and side effects of varying IT fentanyl doses (pruritis, hypotension).

## Methods

Due to the quality improvement nature of our study, IRB approval was exempted by the Human Subject Division of the University of Washington (HSD – 00001072, Approval January 19, 2017) and informed consent was waived to retrospectively analyze the de-identified electronic medical records of all the patients who received a combined spinal epidural (CSE) procedure for labor analgesia at the University of Washington Medical Center Labor and Delivery ward from May 2016 through December 2016 were included.

Our standard CSE spinal dose consists of 1 mL of 0.25% isobaric bupivacaine with added fentanyl. A standard IT fentanyl dose was recommended, while allowing clinical discretion for providers to change the dose if medically indicated. Providers were asked to use IT fentanyl doses less than 25 mcg given the reported risk for NRFHRC. The recommended IT fentanyl dose changed bimonthly ranging from 2.5 mcg, to 5 mcg, then 10 mcg, and 15 mcg in the last two months. The standard medical and nursing management of these patients did not vary otherwise besides what was described above.

Data was collected from the electronic medical record (ORCA, Cerner, North Kansas City, MO) by the Perioperative & Pain initiatives in Quality Safety Outcome Centre (PPiQSO, pronounced “Picasso”). PPiQSO is a division of the Department of Anesthesiology and Pain Medicine tasked to improve perioperative patient outcomes using informatics and technology. The following data variables were collected:

- Patient demographics: age, height, weight (BMI was calculated from the measured height and weight), gravidity, parity, and gestational age.
- Labor outcomes information: if oxytocin was used to augment labor, use of tocolytics (nitroglycerine or terbutaline), and need for emergent cesarean delivery within 20 minutes of CSE placement.
- Presence of prolonged NRFHRC within 20 minutes after CSE (defined as fetal heart rate deceleration lasting >2 minutes). Tracings are interpreted in real-time by trained labor and delivery nurses, who discuss abnormal findings with the team of obstetricians on duty, and then proceed to enter a description of the deceleration in the electronic medical record.
- Anesthetic data: IT fentanyl dose, 11 point VAPS (ranging from 0 = no pain to 10 = maximal pain) immediately before CSE placement, VAPS within 30 minutes after CSE placement, baseline noninvasive blood pressure (NIBP) (defined as pre-procedural NIBP), lowest NIBP within 60 minutes after CSE, vasopressor (phenylephrine or ephedrine) administration to treat hypotension (defined as decrease in mean arterial pressure (MAP) >20% from pre-procedural NIBP), and nalbuphine or diphenhydramine therapy for pruritus.

Patients with extremes in height and weight (height <125 cm or >200 cm, weight <40 kg or >300 kg) were excluded from the analysis to minimize the effect of those confounders.

All data analysis was performed using Microsoft Excel (Microsoft, Redmond, WA). Demographic variables were all continuous, and thus, the mean was analyzed. Most of the labor variables were categorical, binary variables analyzed as percentages. The exception to this is VAPS difference before-CSE minus after-CSE, which is a continuous variable. For the VAPS difference, we analyzed the mean and the 95% confidence interval.

Single factor ANOVA was used to assess the statistical significance of the means for continuous variables such as the demographic variables and the VAPS difference, and Pearson’s chi-squared test was used to assess the statistical significance of categorical variables such as the incidence of NRFHRC (defined as the number of cases with NRFHRC divided by the total number of cases analyzed). In spite of multiple variable comparisons, post hoc analysis with methods like the Bonferroni correction or Tukey’s test of additivity were not performed because we looked at all of the variables individually. We did not intend to draw any conclusions about the groups as a whole based on the multiple variables. We defined statistical significance as a p-value < 0.05.

## Results

A total of 555 records were reviewed. The data was organized into groups according to the CSE IT fentanyl dose: 2.5 mcg, 5 mcg, 10 mcg, and 15 mcg. There were 135, 129, 158, and 131 patients in each group, respectively. The demographic data are summarized in Table 1. There was no significant difference in demographic data between the four groups.

**Table 1:** Patient demographics. “n” is the number of subjects per group. Values are mean (SD).

	Intrathecal fentanyl dose (mcg)				
	
	2.5	5	10	15	p-value
	(n=135)	(n=129)	(n=158)	(n=131)	
**Age; years**	30 (6)	31 (6)	31(5)	30 (5)	0.25
**BMI; kg.m^-2^**	32 (7)	31 (7)	31 (6)	31 (6)	0.60
**Gestational age; weeks**	38 (4)	38 (4)	38 (3)	38 (2)	0.42

The labor and anesthetic variables are summarized in Table 2. Since only a subset of each data group had complete VAPS data, the number of subjects in each group is listed in the table. The VAPS variable data was mined from non-compulsory nurse charting notes, thus some CSE records had either incomplete pre- or post-CSE VAPS, or scores entered greater than 30 minutes before or after the CSE. Incomplete records were excluded for the VAPS variable comparison only. The other labor and anesthetic variables were complete for the 555 records analyzed.

**Table 2:** Labor and anesthetic variables. Values are mean (95% CI) or number (proportion). “n” is the number of subjects per group. “n” is the same for all rows *except* for row 2 “VAPS* difference before-CSE^†^ minus after-CSE.”

		Intrathecal fentanyl dose (mcg)			
	
	2.5	5	10	15	
	(n=135)	(n=129)	(n=158)	(n=131)	p value
**Oxytocin CSE administration prior to**	47 (35%)	37 (29%)	52 (33%)	56 (43%)	0.18
**VAPS difference before-CSE**	5.3	5.6	5.3	6.7	
**minus after-CSE**	(4.4–6.1)	(4.9–6.4)	(4.5–6.1)	(6.0–7.4)	0.07
	n=60	n=67	n=69	n=68	
**Use of nalbuphine**	1 (0.7%)	4 (3.1%)	5 (3.1%)	7 (5.3%)	0.99
**Use of diphenhydramine**	1 (0.7%)	0	1 (0.6%)	0	0.99
**Decrease in MAP‡ > 20% after CSE**	50 (37%)	50 (39%)	62 (39%)	45 (34%)	0.24
**Use of phenylephrine**	3 (2.2%)	1 (0.8%)	4 (2.5%)	3 (2.3%)	0.83
**Use of ephedrine**	2 (1.5%)	2 (1.6%)	8 (5.0%)	1 (0.8%)	0.12
**NRFHRC^§^**	6 (4.4%)	3 (2.3%)	12 (7.6%)	4 (3.0%)	0.11
**Use of nitroglycerine**	1 (0.7%)	0	2 (1.3%)	0	0.49
**Use of terbutaline**	0	0	1 (0.6%)	0	0.61
**Emergent minutes after cesarean CSE delivery 30**	0	0	2 (1.3%)	0	0.25

* VAPS, 11-point Visual Analog Pain Score; † CSE, Combined Spinal Epidural Labor Analgesia; ‡ MAP, Mean Arterial Pressure; **§** NRFHRC, Non-reassuring Fetal Heart Rate Changes

None of the labor or anesthetic variables reached statistical significance. The incidence of NRFHRC of the entire cohort was 4.5%. The incidence did not show any trend to increase with increasing fentanyl dose, nor were the differences between groups significant. Two women in the 10-mcg fentanyl group required emergent cesarean delivery that was potentially related to the CSE, compromising 0.36% of all patients. The “emergent cesarean delivery” variable was defined as the incidence of cesarean delivery with “Anesthesia Start” time within 20 minutes of CSE placement. These two patients did not receive nitroglycerin, terbutaline, phenylephrine, or ephedrine. Only one of the two was noted to have a NRFHRC. The overall rate of cesarean delivery in the entire cohort was 25.2%.

Although pain reduction after CSE was similar regardless of the IT fentanyl dose, this reduction in VAPS appeared adequate, with reported scores decreasing by 5–7 points on the 11-point scale. There was a trend to slightly greater pain reduction at higher doses of IT fentanyl. However, the 95% confidence intervals of the VAPS reduction for 2.5 and 15 mcg of IT fentanyl slightly overlapped. See Figure 1 for details.

The incidence of itching requiring treatment with nalbuphine or diphenhydramine was very low, and not significantly different between the groups. At most, 5.3% of patients who received 15 mcg IT fentanyl required treatment for itching.

A larger fraction of women in each group, 34–39%, experienced decrease in MAP > 20% from baseline. Only a small number required treatment of hypotension with a combined incidence of 4.3%. Phenylephrine was given to 0.8–2.5%, and 0.8–5.0% ephedrine. The incidence of hypotension and percentage of women requiring pharmacologic treatment of hypotension was not significantly different between the groups.

## Discussion

Prompted by a small series of non-reassuring fetal heart changes after CSE placement for labor on our obstetric unit, as part of a quality improvement initiative, we examined the effect of varying doses of IT fentanyl (2.5, 5, 10, and 15 mcg) added to 2.5 mg isobaric bupivacaine in CSEs on maternal and fetal variables. We did not observe a difference in our primary outcome, the incidence of NRFHRC, or any of our secondary outcomes: emergent cesarean delivery within 20 minutes of CSE, reported intrapartum pain scores, pruritus, or hypotension.

The etiology of NRFHRC after IT opioids has been attributed to rapid onset of analgesia causing a decrease in stress hormones such as adrenaline and noradrenaline. Stress hormones are associated with decreased uterine activity.[25] Thus, the acute reduction of circulating stress hormones by analgesia may lead to uterine hyperactivity and even tetanic contraction. This can cause placental hypoperfusion, followed by NRFHRC.[26,27] Based on this theory, the labor anesthetic that results in the most rapid onset of pain relief would have the highest risk of causing NRFHRC, but the literature does not universally support this finding. Some studies have shown that intrathecal opioids do cause more NRFHRC than epidural analgesia, while others have shown equal rates. ^[19,23,28,29]^ A Cochrane review comparing CSEs and epidurals did not address NRFHRC.[15] Cascio et al. measured venous epinephrine levels after neuraxial anesthesia with IT fentanyl and epidural 1.5% lidocaine and found that both lowered venous epinephrine levels to a similar degree, though fentanyl lowered epinephrine levels slightly faster.[30] Studies of CSEs with IT fentanyl from 5–45 mcg and 0–25 mcg did not show differences in the rates of NRFHRC.[18,22] We did not find that varying the CSE IT opioid dose resulted in different rates of NRFHRC.

There are other theories as to why IT opioids may contribute to NRFHRC. One study examined whether IT opioids modulated endogenous oxytocin release and found no relationship.[19] Another study examined hemodynamic effects of IT fentanyl and found that the periods of hypotension that followed were brief and not associated with NRFHRC.[31] Nicolet et al. postulated that factors other than regional analgesia technique could also be related to NRFHRC. They found that older maternal age and higher pain scores prior to labor analgesia were the only independent predictors of NRFHRC on multivariable analysis.[29]

The incidence of NRFHRC in our whole cohort was 4.5% and did not differ between groups. This is on par or lower than the other reported incidences. In a review by Mardirosoff, the incidence of NRFHRC was found to be 7.3–7.7% with large proportion of CSEs using doses above 25 mcg IT fentanyl. [12] Another review found the incidence to be 4–15%.[19] One study found a much higher incidence of NRFHRC of 24% of patients who had CSEs with 7.5 mcg IT sufentanil, and in 12% of patients with CSEs with 1.5 mcg IT sufentanil.[32] Using the IT potency comparison of 4.4:1 for sufentanil : fentanyl, 7.5 mcg of IT sufentanil equates to 33 mcg of IT fentanyl.[33]

In spite of the few cases of NRFHRC after CSE in our study, the overall rate of unplanned cesarean delivery in our study was 25.2%, and comparable with the overall US cesarean delivery rate of roughly 33%.[34] We have a robust treatment algorithm in place in the event of NRFHRC after CSEs that includes left uterine displacement to avoid supine hypotension syndrome, hemodynamic stabilization with vasopressors, tocolysis with intravenous nitroglycerine or intramuscular terbutaline, and supplemental maternal oxygenation. Of note, only two of those cesarean deliveries happened within 20 minutes after CSE placement. Our data is not adequate to determine if the IT fentanyl contributed to the need for those cesarean deliveries though neither patient received tocolytics or vasopressors in the time period between the initiation of labor analgesia and conversion to surgical anesthesia.

We found that varying IT fentanyl doses in this dose range did not cause differences in VAPS reduction after CSE. Wong conducted a prospective study with similar doses of IT fentanyl as used in our population (0, 5, 10, 15, 20, 25 mcg). As in our study, analgesia was similar in groups who received up to 10 mcg of IT fentanyl. Doses above 15 mcg were superior to groups 0 mcg, 5 mcg, and 10 mcg, but we do not routinely use these higher doses in our practice. All the groups that received any fentanyl were shown to experience lasting analgesia beyond 15 minutes after application.[18] Some previous studies did show a difference in pain control with varying doses even at these lower levels. Palmer saw analgesia duration increase from doses of 5 mcg IT fentanyl up to 25 mcg, and they found the median effective dose to be 14 mcg.[22] Lo found a significant difference in VAPS at 30 minutes post CSE between women who received 2.5 mg IT bupivacaine with 10 mcg of IT fentanyl and women who had CSEs with 2.5 mg IT bupivacaine without IT fentanyl.[35]

Pruritis is a known to occur at higher rates after IT opioids as compared to epidural labor analgesia.^[12,13, 14, 15]^ The incidence of severe itching requiring treatment with either nalbuphine or diphenhydramine was 3.4% in our whole cohort, and not statistically significant for either drug between the groups. This is on par with other studies. Palmer found that the incidence of pruritus was the same regardless of IT fentanyl dose from 5 to 45 mcg, although patients rated their severity of pruritus to be lower in the 5 to 10 mcg IT fentanyl range. The only patients requiring treatment for pruritus had IT fentanyl doses > 15 mcg.[22] Collis found that 3% of patients who received 25 mcg IT fentanyl via CSEs required pruritis treatment with naloxone. Generally, the reported incidence of itching in the literature is higher, for example, 42% in the Collis study and 52% in the Mardirosoff review, however, severe itching requiring treatment is much lower.[3,12]

Hypotension is a known side effect of all types of neuraxial labor analgesia.[15] Our incidence of hypotension was 34–39% and not significant between the groups. Depending on the definition of hypotension and study medications, incidence in the literature varies up to 50% of patients.[31] Not all hypotension requires treatment though. The overall proportion of our patients who received a dose of vasopressors was low at 4.3%. One meta-analysis found an increased incidence of hypotension when an IT opioid bolus was given along with epidural opioid as maintenance analgesia, suggesting that hypotension is dose-dependent.[28] Our IT fentanyl dose may have been too low for this effect to have been seen in the 60 minutes after CSE initiation.

In light of prior findings, the design of this retrospective analysis may not be ideal to illuminate the fine difference in pain control at these lower varying IT fentanyl doses. The diferences in pain control may be better determined by measuring the duration of analgesia provided by the spinal dose, whereas the pain scores after neuraxial placement we measured were reported anywhere from 0 to 30 minutes post CSE.

This study is limited by its retrospective nature and dependence on the robustness of the electronic medical record. Retrospective analyses are often subject to biases that could have affected the doses chosen for IT fentanyl, and the subsequent patient grouping. The robustness of the electronic medical record limited the completeness of the records we could obtain, which afected our power and measurement precision. For example, only a subset of the records had complete VAPS records before and after CSE limiting our ability to reach statistical significance with our measurements of that variable. Also, those complete records were not precisely time stamped relative to the CSE, and not recorded frequently enough so that we could make a determination about the duration of analgesia after CSEs with varying IT fentanyl doses. Furthermore, the assessment of pruritus was not uniformly recorded from patient to patient, so we could only assess the incidence of severe pruritus by incidence of nalbuphine or diphenhydramine drug delivery.

In summary, a small series of NRFHRC after CSE labor analgesia prompted us to perform a large-scale, quality improvement audit of our practice. After varying IT fentanyl doses within what the literature has shown to be safe and corresponding to normal practice parameters, we performed a retrospective, de-identified, chart analysis to study the effect on several labor variables. We found no significant diference in the incidence of NRFHRC, emergent cesarean delivery 20 minutes post-CSE, reported pain score reduction, presence of severe pruritus requiring treatment, or hypotension. We reaffirm that performing CSEs for labor with IT fentanyl less than 15 mcg is a safe and effective practice.
